# Overview of adjuvant radiotherapy on survival, failure pattern and toxicity in stage I to II endometrial carcinoma: a long-term multi-institutional analysis in China

**DOI:** 10.1186/s12885-022-09343-4

**Published:** 2022-03-14

**Authors:** Wenhui Wang, Tiejun Wang, Zi Liu, Jianli He, Xiaoge Sun, Wei Zhong, Fengjv Zhao, Xiaomei Li, Sha Li, Hong Zhu, Zhanshu Ma, Ke Hu, Fuquan Zhang, Xiaorong Hou, Lichun Wei, Lijuan Zou

**Affiliations:** 1 Department of Radiation Oncology, Peking Union Medical College Hospital Chinese Academy of Medical Sciences & Peking Union Medical College, No. 1 Shuaifuyuan Wangfujing Dongcheng District, Beijing, People’s Republic of China; 2 Department of Radiation Oncology, The second hospital Affiliated by Jilin University, Changchun, People’s Republic of China; 3 Department of Radiation Oncology, First Affiliated Hospital of Xi’an Jiaotong University, Xi’an, People’s Republic of China; 4 Department of Radiation Oncology, The General Hospital of Ningxia Medical University, Yinchuan, Ningxia People’s Republic of China; 5 Department of Radiation Oncology, The Affiliated Hospital of Inner Mongolia Medical University, Hohhot, Inner Mongolia People’s Republic of China; 6 Gynaecological Oncology Radiotherapy, The Affiliated Cancer Hospital of Xinjiang Medical University, Urumqi, People’s Republic of China; 7 Department of Radiation Oncology, Gansu Provincial Cancer Hospital, Lanzhou, Gansu People’s Republic of China; 8 Department of Radiation Oncology, Peking University First Hospital, Beijing, People’s Republic of China; 9 Department of Radiation Oncology, The 940th Hospital of Joint Logistics Support force of Chinesc People’s Liberation Army, Lanzhou, Gansu People’s Republic of China; 10 Department of Radiation Oncology, Xiangya Hospital Central South University, Changsha, Hunan People’s Republic of China; 11 Department of Radiation Oncology, Affiliated Hospital of Chi feng University, Chifeng, Inner Mongolia People’s Republic of China; 12 Department of Radiation Oncology, Xijing Hospital, Air Force Medical University of PLA (the Fourth Military Medical University), Xi’an, People’s Republic of China; 13 Department of Radiation Oncology, The Second Hospital of Dalian Medical University, Dalian, People’s Republic of China

**Keywords:** Endometrial neoplasms, Radiotherapy, Survival analysis, Failure pattern, Toxicity

## Abstract

**Background:**

This research aimed to provide an overview of the impact of adjuvant vaginal brachytherapy (VBT) and external beam pelvic radiotherapy (EBRT) with or without VBT on survival in stage I to II EC patients in China from a long-term multi-institutional analysis.

**Methods:**

We retrospectively analyzed stage I to II EC patients from 13 institutions treated between 2003 and 2015. All patients underwent surgical staging and received adjuvant RT. Patients were divided into groups of low-risk (LR), intermediate-risk (IR), high-intermediate-risk (HIR) and high-risk (HR). Survival statistics, failure pattern, and toxicity of different radiation modalities in different risk groups were analyzed.

**Results:**

A total of 1048 patients were included. HR disease represented 27.6%, HIR 17.7%, IR 27.7% and LR 27.1%, respectively. Endometrioid adenocarcinoma (EAC) and non-endometrioid carcinoma (NEC) accounted for 92.8 and 7.2%. A total of 474 patients received VBT alone and 574 patients received EBRT with or without VBT.

As for EAC patients, the 5-year overall survival (OS), disease-free survival (DFS), local recurrence-free survival (LRFS), and distant metastasis-free survival (DMFS) rate was: 94.6, 90.4, 93.0 and 91.6%, respectively. For LR patients, EBRT (with or without VBT) seemed to be a risk factor. With the higher risk category, the survival benefit of EBRT gradually became remarkable. EBRT (with or without VBT) significantly increased DFS, LRFS and DMFS compared to VBT alone in the HR group (*p* < 0.05). Distant metastasis was the main failure pattern for all risk groups. As for NEC patients, the 5-year OS, DFS, LRFS and DMFS rate was: 93.4, 87.2, 91.7 and 89.3%, respectively. As for toxicity, EBRT (with or without VBT) significantly increased the incidence of grade 1–2 gastrointestinal, urinary, and hematological toxicity.

**Conclusions:**

For stage I to II EC patients, EAC accounted for the majority and had better prognosis than NEC. For EAC patients, VBT alone resulted in comparable survival to EBRT in the LR, IR and HIR groups, while EBRT significantly increased survival in the HR group. EBRT had higher rate of toxicity than VBT.

## Background

In China, uterine tumor is the second-most common cancer of the female reproductive system. Data from the National Central Cancer Registry of China indicated that the number of new diagnoses of uterine tumors was 634,000 in 2015, most of which were endometrial cancer (EC) [1]. Considering the great population, it is of high importance to explore and cure patients with EC. The initial management for uterine-confined EC is total abdominal hysterectomy and bilateral salpingo-oophorectomy with or without lymph node dissection. Adjuvant radiotherapy (RT), including external beam pelvic radiotherapy (EBRT) and/or vaginal brachytherapy (VBT), demonstrates good prognosis and is commonly recommended based on risk classifications [2–4].

There are currently several risk stratification criteria in clinic. In 2014, the ESMO (European Society for Medical Oncology) - ESGO (European Society of Gynecological Oncology) - ESTRO (European Society for Radiotherapy & Oncology) consensus was devised and classified EC into low-risk (LR), intermediate-risk (IR), high-intermediate-risk (HIR), and high-risk (HR) groups [3], which is widely used nowadays. In 2021, the ESGO- ESTRO, and the European Society of Pathology (ESP) jointly updated these evidence-based guidelines and covered molecular characteristics to devise the new ESGO/ESTRO/ESP guidelines for the management of patients with EC [4].

As the World Health Organization (WHO) classified, EC included endometrioid adenocarcinoma (EAC) and non-endometrioid carcinoma (NEC) [5]. Many randomized trials have been conducted, and abundant survival data of EC across the world was reported. Compared to EAC, most research showed that NEC had been associated with significantly poorer survival rates [6–8]. However, there is no comprehensive study on survival outcomes in China. The purpose of this study was to thoroughly investigate the effect of different radiation modalities on survival, failure pattern, and toxicity according to the ESMO-ESGO-ESTRO risk group consensus (2014) in a large-scale, real-world cohort of patients with stage I to II EC in a multi-institutional setting.

## Methods

### Patient selection and eligibility criteria

We retrospectively evaluated the clinical data of patients with stage I to II EC from 13 institutions in China who were treated between Jan. 2003 and Dec. 2015. All patients underwent surgery and adjuvant RT with or without adjuvant chemotherapy. Patients with the following clinical scenarios were excluded: previous chemotherapy or RT, without adjuvant RT, stage III to IV disease, missing data, and palliative surgery. All patients diagnosed before 2009 were restaged according to the International Federation of Gynecology and Obstetrics (FIGO) (2009) staging system. Pathological type was determined by the WHO Classification of tumors of the uterine corpus (2014). Patients were divided into low-risk (LR), intermediate-risk (IR), high-intermediate risk (HIR), and high-risk (HR) groups according to ESMO-ESGO-ESTRO risk classification (2014). LR group was defined as: stage I endometrioid, grade 1–2, < 50% myometrial invasion (MI), and lympho-vascular space invasion (LVSI) negative. IR group was defined as: stage I endometrioid, grade 1–2, ≥50% MI, and LVSI negative. HIR group was defined as: stage I endometrioid, grade 3, < 50%MI, regardless of LVSI status; or stage I endometrioid, grade 1–2, LVSI unequivocally positive, regardless of depth of invasion. HR group was defined as: stage I endometrioid, grade 3, ≥50% MI; or stage II endometrioid, or stage I to II non-endometrioid. As stage III to IV disease were not included, HR disease only referred to HR stage I to II disease. The clinical trial ID of the study is ChiCTR-PRC-17010712 (http://www.chictr.org.cn/index.aspx), which was approved by the Institutional Review Board of Peking Union Medical College Hospital (N0. S-K139).

### Treatment approaches

All patients underwent a total hysterectomy with bilateral salpingo-oophorectomy. Lymphadenectomy was frequently performed among which sentinel lymphadenectomy was used for selected patients. Radiotherapy was administered to all patients, and the pattern of radiation modality was based on patients’ pathological findings, physical condition, doctors’ preference, and patients’ preference after counseling by treating doctor. EBRT was delivered to the pelvic lymphatic drainage area and the upper part of the vaginal stump. High-dose rate VBT was delivered either as postoperative monotherapy or as a boost to EBRT. Intravenous concurrent or sequential adjuvant chemotherapy consisted of carboplatin/paclitaxel, cisplatin/doxorubicin, cisplatin/doxorubicin/paclitaxel, etc. The indication of chemotherapy was based on the doctor’s recommendations, pathological findings, intraoperative conditions, and the patient’s physical condition. In this study, chemotherapy was often recommended for patients with NEC and HIR, HR NAC. Radiation toxicities were evaluated by the Common Terminology Criteria for Adverse Events Version 4.0 (CTCAE 4.0) [9].

### Study endpoints

In this research, we would report radiation modalities among risk groups, survival outcome, failure pattern, and toxicity of the study population. Survival endpoints were defined as overall survival (OS, the time from surgery to death or last follow-up), disease-free survival (DFS, the time from surgery to treatment failure or death or last follow-up), local recurrence-free survival (LRFS, the time from surgery to locoregional failure or death or last follow-up) and distant metastasis-free survival (DMFS, the time from surgery to distant metastasis or death or last follow-up). Failure pattern was classified as local, regional, and distant relapse which were calculated between different risk groups under different radiation modalities. Acute and chronic adverse effects were compared between different radiation modalities.

### Data analysis

Data analysis was performed using SPSS statistical software (version 25.0; SPSS Inc., Chicago, IL). The chi-square test was used to assess differences among categorical variables. Student-t test was used to assess differences among normal distribution continuous variables. The Kaplan-Meier method was used to calculate survival data. Log-rank test was performed to determine differences between groups and *p*-value of < 0.05 was considered statistically significant.

## Results

### Study population

A total of 1178 stage I to II patients who received postoperative RT between Jan. 2003 and Dec. 2015 across 13 Chinese institutions were reviewed. Among them, 1048 patients with intact data were included. Clinical and pathological details were listed in Table 1.

**Table 1:** Baseline Clinical Characteristics for all Patients Treated from 2003 to 2015

	Patients (***N*** = 1048)						
	Total (***n*** = 1048)		Endometrioid Adenocarcinoma (***n*** = 973)		Nonendometrioid Carcinoma (***n*** = 75)		***p***-value
**Clinical Characteristic**	No.	%	No.	%^b^	No.	%^b^	
**Age, years**							0.129
Mean	56.1		56.0		57.6		
Range	23–86		23–86		33–79		
**Lymphadenectomy**							0.010
No	319	30.4	306	31.4	13	17.3	
Yes	729	69.6	667	68.6	62	82.7	
Median Number.	21.0		21.0		18.0		0.009
Range	1–65		1–65		1–42		
**Stage (FIGO 2009)**							0.012
IA	474	45.2	428	44.0	46	61.3	
IB	426	40.7	406	41.7	20	26.7	
II	148	14.1	139	14.3	9	12.0	
**Diameter**							0.630
< 2 cm	111	10.6	102	16.1	9	18.8	
≥ 2 cm	571	54.5	532	83.9	39	81.3	
Missing	366	34.9					
**Grade**^a^							
G1	327	33.6	327	33.6	/	/	
G2	447	45.9	447	45.9	/	/	
G3	191	19.7	191	19.7	/	/	
Missing	8	0.8	8	0.8	/	/	
**Myometrial invasion**							0.012
< 1/2	535	51.0	486	50.4	49	65.3	
≥ 1/2	505	48.2	479	49.6	26	34.7	
Missing	8	0.8					
**Invasion of lower uterine segment**							0.080
No	746	71.2	686	70.5	60	80.0	
Yes	302	28.8	287	29.5	15	20.0	
**LVSI**							0.601
Present	173	16.5	159	16.3	14	18.7	
Absent	875	83.5	814	83.7	61	81.3	
**Chemotherapy**							0.000
No	820	78.2	786	80.8	34	45.3	
Yes	228	21.8	187	19.2	41	54.7	

*Abbreviations*: *FIGO* International Federation of Gynecology and Obstetrics, *LVSI* Lympho-vascular Space Invasion

^a^only for endometrioid adenocarcinoma

^b^When compared between patients with endometrioid adenocarcinoma and nonendometrioid carcinoma, only the percnet of known numbers were calculated

The main pathological type was endometrioid adenocarcinoma (EAC), while non-endometrioid carcinoma (NEC) accounted for only 7.2%, including mixed cell carcinoma (*n* = 39), serous carcinoma (*n* = 18), clear cell carcinoma (*n* = 11), undifferentiated carcinoma (*n* = 6), and dedifferentiated carcinoma (*n* = 1). For EAC, the degree of differentiation was mainly grade 2. Compared with patients with EAC, patients with NEC had significantly milder pathological characteristics, that was, a higher proportion of stage IA disease (*p* = 0.012) and superficial MI (*p* = 0.012). From the perspective of treatment, patients with NEC received a higher rate of lymphadenectomy (*p* = 0.010) and chemotherapy (*p* = 0.000).

Lymphadenectomy was achieved in 69.6% of all patients. Chemotherapy was administered to 21.8%. As to adjuvant RT, the dose-fractionation schedules were prescribed according to the guidelines and slightly adjusted among the centers. For EBRT, the most common dose-fractionation schedule was 50 Gy in 25 fractions. As for radiotherapy technique, computer tomography-based intensity modulated RT accounted for the majority (45.5%), followed by three-dimensional conformal RT modality (26.0%) and four-field box technique (28.5%). For VBT, the most common dose-fractionation schedule for VBT alone was 6 fractions 5 Gy each, and for VBT as a boost was 2 fractions 5 Gy each.

### Risk categories and RT modalities

**Fig. 1:**
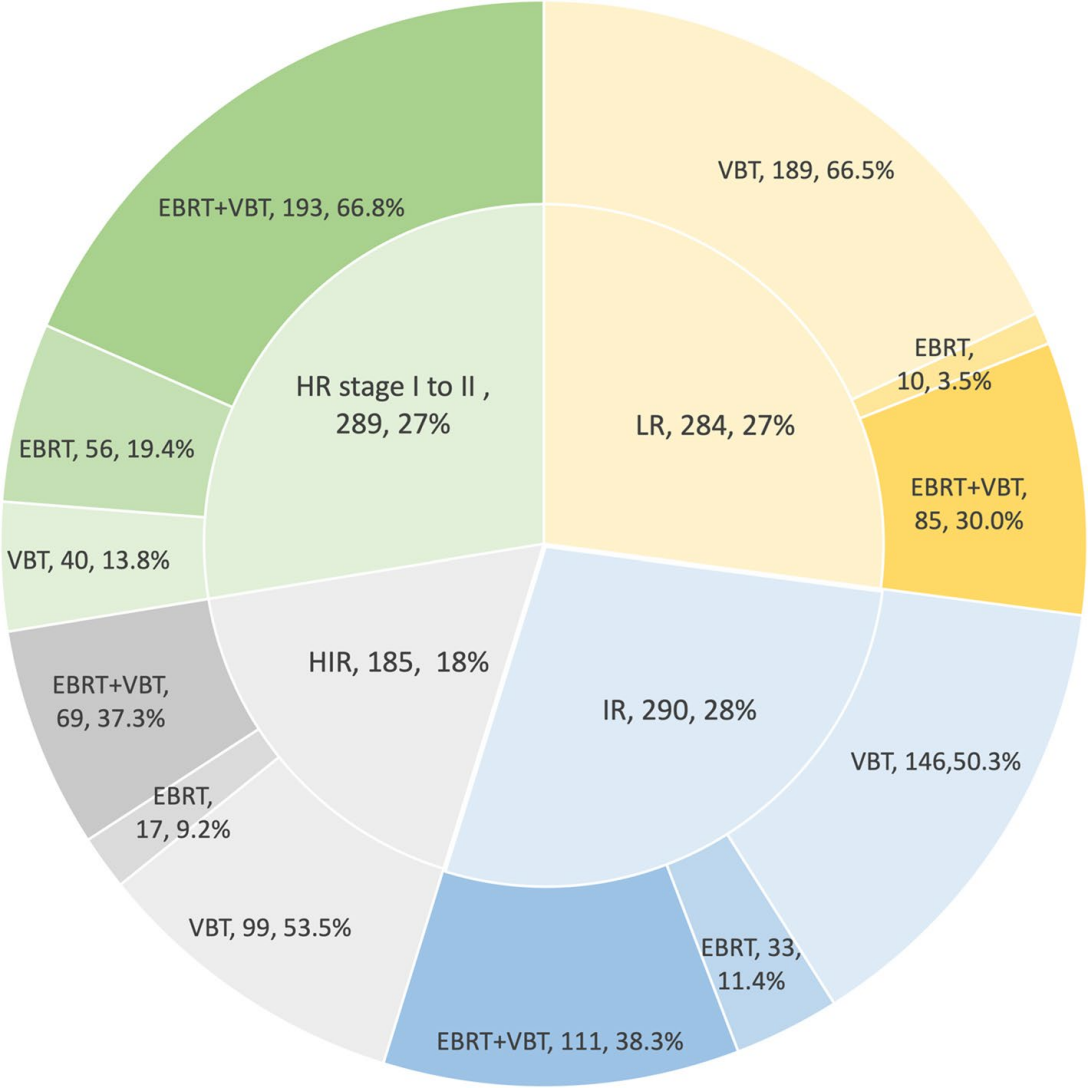
Distribution of radiotherapy modalities among different risk groups

As for the risk stratification groups, LR disease represented 27.1% (*n* = 284), IR 27.6% (*n* = 290), HIR 17.7% (*n* = 185), and HR stage I and II disease represented 27.6% (*n* = 289) of the cohort. For radiation modality, 474 patients received VBT alone (45.2%), and 574 patients received EBRT (specifically, 458 patients received EBRT with VBT, and 116 patients received EBRT without VBT). With the higher risk category, the proportion of VBT alone decreased from 66.5% in the LR group to 13.8% in the HR stage I and II group, while the proportion of EBRT with VBT increased from 30.0% in the LR group to 66.8% in the HR stage I and II group (Fig. 1).

### Survival

A total of 973 patients with EAC and 75 patients with NEC were included in this study. Due to the different biological characteristics, survival and failure pattern analysis were performed for EAC and NEC, respectively.

For patients with EAC, the median follow-up time was 56.0 months (range: 2 to 204 months). The 5-year OS, DFS, LRFS and DMFS rate was: 94.6, 90.4, 93.0, and 91.6%, respectively.

**Fig. 2:**
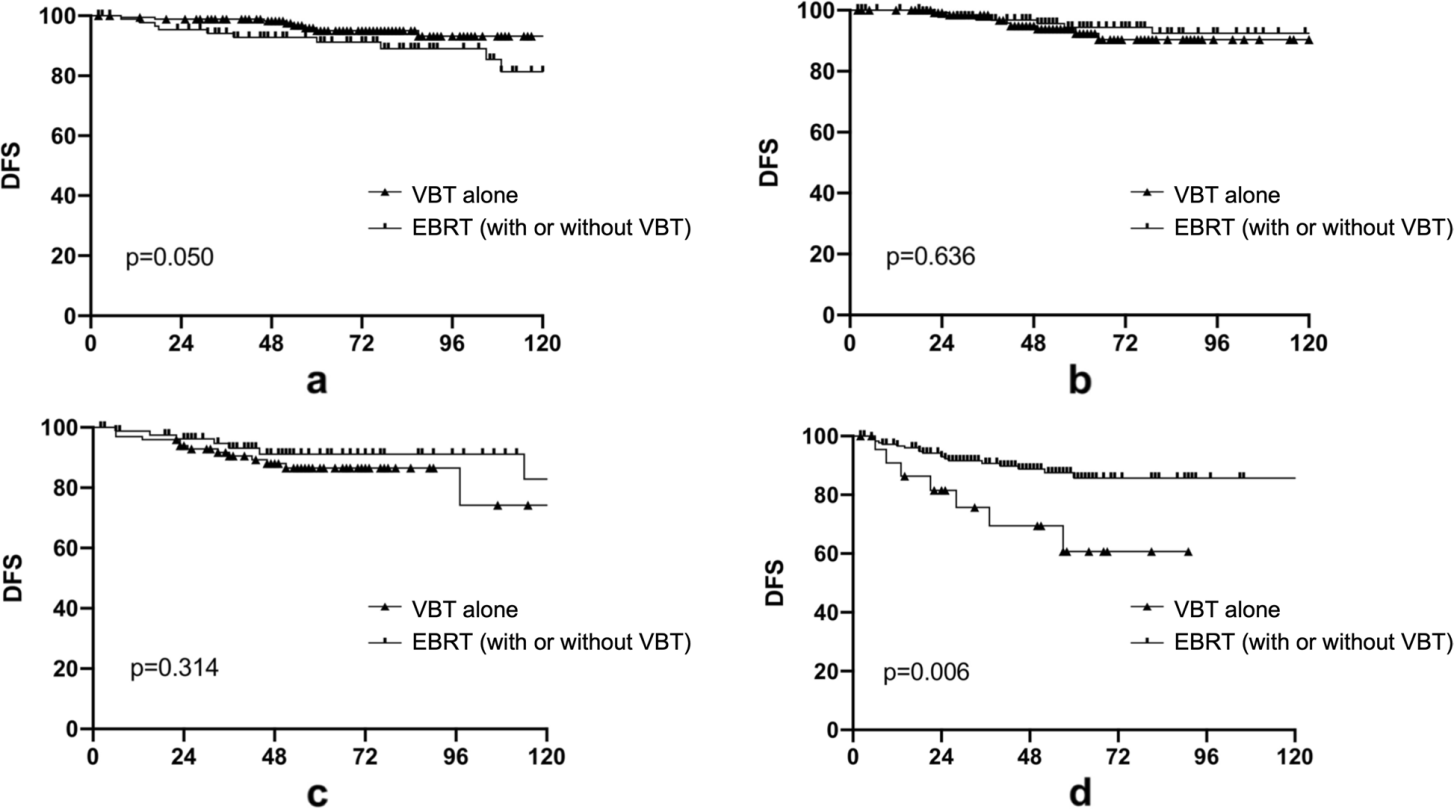
Effect of VBT alone or EBRT (with or without VBT) on disease-free survival (DFS) for patients among different risk groups. **a** Low-risk; **b** Intermediate -risk; **c** High-intermediate-risk; **d** High-risk

**Fig. 3:**
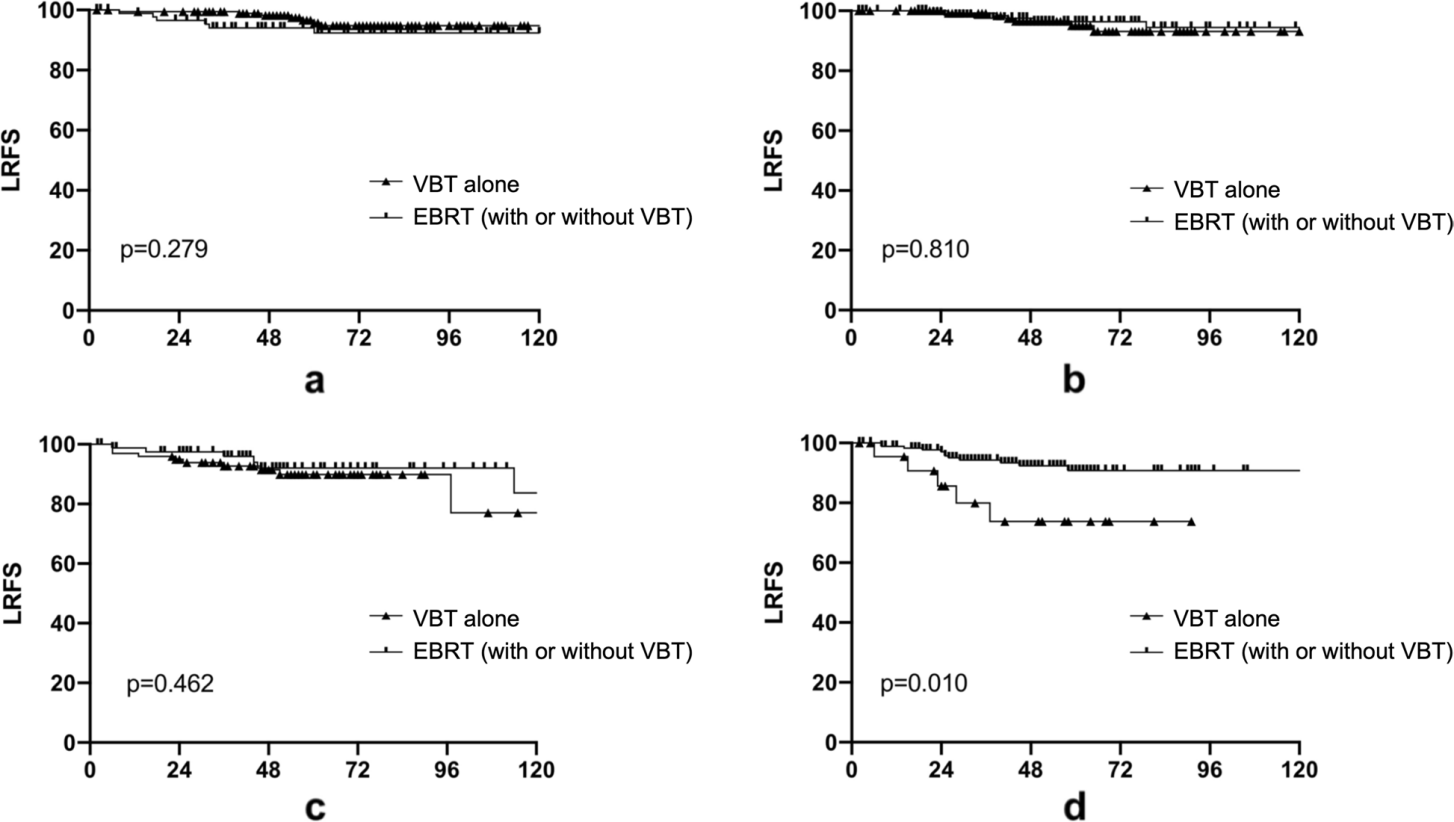
Effect of VBT alone or EBRT (with or without VBT) on local-regional failure free survival (LRFS) for patients among different risk groups. **a** Low-risk; **b** Intermediate -risk; **c** High-intermediate-risk; **d** High-risk

We compared the effect on survival of EBRT (with or without VBT) versus VBT alone for patients with different risk stratifications (Figs. 2, Fig. 3). For LR patients, EBRT (with or without VBT) seemed to be a risk factor. DFS (2-year DFS: 95.4% vs. 98.9%, *P* = 0.05) was marginally significantly lower in patients who received EBRT (with or without VBT) than those who were treated with VBT alone. OS (2-year rate: 100.0% vs. 100.0%, *p* = 0.241), LRFS (2-year rate: 96.5% vs. 99.4%, *p* = 0.279), and DMFS (2-year rate: 96.5% vs. 98.9%, *p* = 0.096) rates were slightly lower for patients receiving EBRT (with or without VBT) than those receiving VBT alone. With the increasing risk, the survival benefit of EBRT (with or without VBT) gradually became remarkable. For IR patients, the survival curves of patients receiving EBRT (with or without VBT) and VBT alone overlapped. For HIR patients, EBRT (without or without VBT) increased DFS (2-year DFS: 96.2% vs. 93.9%, *p* = 0.314), although non-statistically significantly, compared with those with VBT alone, and the curves did not overlap. For HR stage I and II patients, DFS (2-year rate: 92.9% vs. 81.6%, *p* = 0.006), LRFS (2-year rate: 97.0% vs. 85.6%, *p* = 0.007) and DMFS (2-year rate: 92.9% vs. 81.3%, *p* = 0.005) rates were significantly higher for patients who received EBRT (without or without VBT) than those who were treated with VBT alone, while OS was marginally increased (2-year OS: 97.0% vs. 90.5%, *p* = 0.059).

In order to exclude the efficacy of chemotherapy on survival, we only included patients who received RT (*n* = 267 in LR group, *n* = 256 in IR group, *n* = 141 in HIR group, *n* = 122 in HR group) in the survival analysis. Conclusions were similar to those above. The survival outcomes of EBRT (with or without VBT) was similar to that of VBT alone in LR, IR and HIR groups. In HR group, EBRT (with and or VBT) significantly improved DFS (2-year DFS: 92.5% vs. 82.4%, *p* = 0.012) and DMFS (2-year DMFS: 92.5% vs. 81.9%, *p* = 0.008), improved LRFS (2-year LRFS: 95.7% vs. 87.8%, *p* = 0.051) with marginal significance. Although OS (2-year OS: 97.6% vs. 87.5%, *p* = 0.149) was not improved, the curve was separated.

**Fig. 4:**
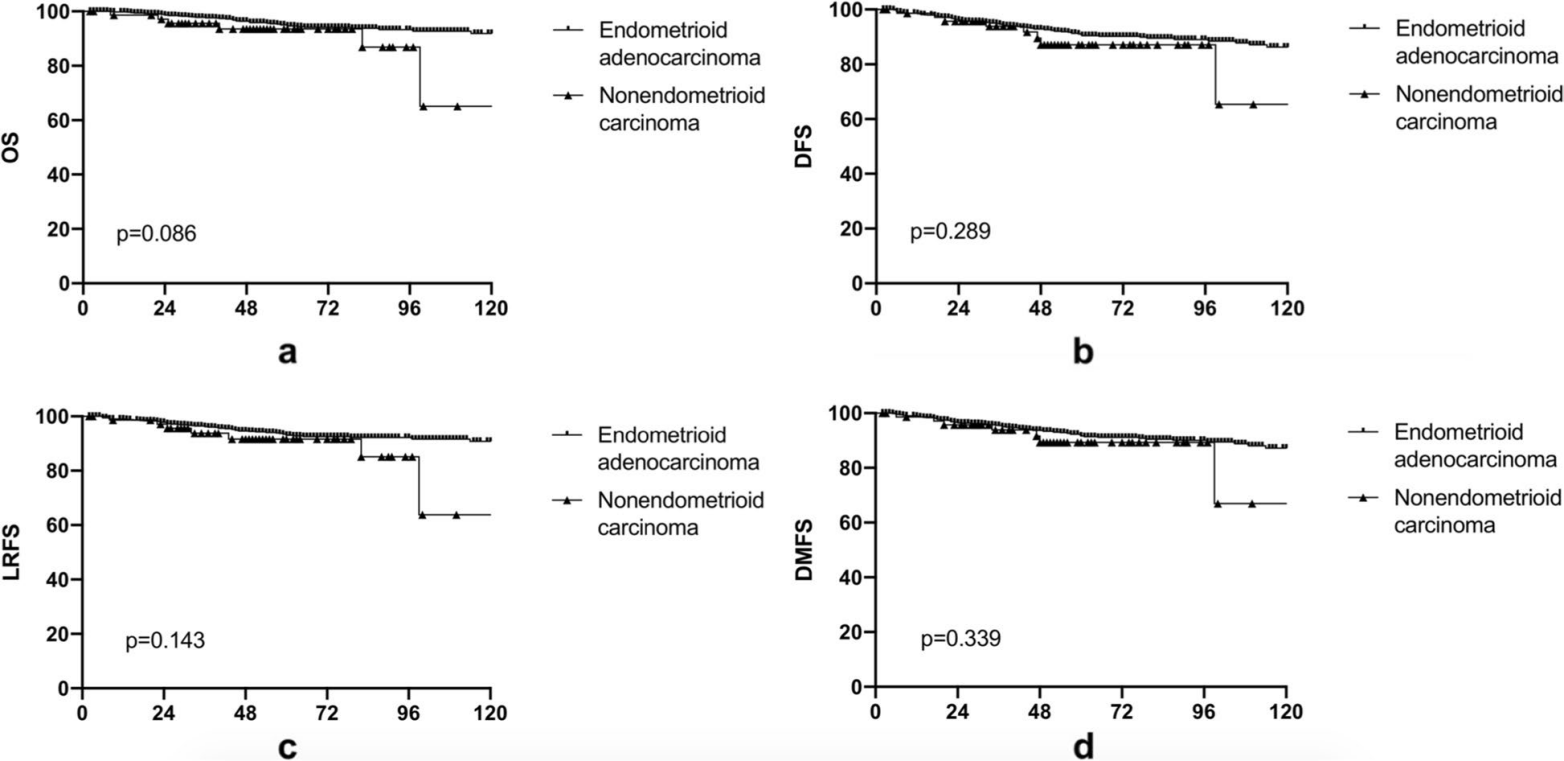
Comparation of endometrioid adenocarcinoma (EAC) and non-endometrioid carcinoma (NEC) on survival outcomes. **a** overall survival (OS); **b** disease-free survival (DFS); **c** local-regional failure free survival (LRFS); **d** distant metastasis failure free survival (DMFS)

As for patients with NEC, the median follow-up time was 49.0 months (range: 2 to 150 months). Compared to patients with EAC, patients with NEC had lower 5-year rates of OS (93.4%), DFS (87.2%), LRFS (91.7%), and DMFS (89.3%). Although there was no statistically significant difference compared to EAC, the survival curves did not cross (Fig. 4).

### Failure pattern

As for the 973 EAC patients, 69 (7.1%) relapsed. Distant metastasis (DM) was the main failure mode in all risk groups with an incidence of 5.9% (*n* = 57). In the HR stage I and II group, compared to VBT alone, EBRT (with or without VBT) significantly reduced the local or regional recurrence rate and decreased -- although non-statistically significant -- the local, regional, or distant failure rate. In the HIR group, for patients treated with EBRT (with or without VBT), the local, regional, or distant failure rate decreased although non-statistically significant. In the LR and IR groups, different RT modes had little effect on different failure sites (Table 2).

**Table 2:** Failure Pattern for EAC Patients Treated with VBT alone or EBRT (with or without VBT) in Different Risk Groups

	Patients (*N* = 973)							
Failure mode	Local No. (%)	***p***	Regional No. (%)	***p***	Local or Regional^a^ No. (%)	***p***	Distant	***p***
**Low-risk** (*N* = 284)		0.215		0.536		0.666		0.195
**VBT alone** (*N* = 189)	1 (0.5)		3 (1.6)		3 (1.6)		6 (3.2)	
**EBRT (with or without VBT)** (*N* = 95)	3 (3.2)		0 (0.0)		3 (3.2)		7 (7.4)	
**Intermediate risk-risk** (*N* = 290)		0.990		0.605		0.433		0.796
**VBT alone** (*N* = 146)	1 (0.7)		1 (0.7)		2 (1.4)		7 (4.8)	
**EBRT (with or without VBT)** (*N* = 144)	2 (1.4)		3 (2.1)		5 (3.5)		6 (4.2)	
**High-intermediate-risk** (*N* = 185)		1.000		0.377		0.249		0.639
**VBT alone** (*N* = 99)	2 (2.0)		6 (6.1)		7 (7.1)		6 (6.1)	
**EBRT (with or without VBT)** (*N* = 86)	1 (1.2)		2 (2.3)		2 (2.3)		3 (3.5)	
**High-risk** (*N* = 214)		0.218		0.178		0.016		0.147
**VBT alone** (*N* = 24)	1 (4.2)		2 (8.3)		7 (7.1)		5 (20.8)	
**EBRT (with or without VBT)** (*N* = 190)	0 (0.0)		3 (1.6)		2 (2.3)		17 (8.9)	

*Note*: ^a^ “Local or Regional” meant relapse at either local or regional site

*Abbreviations*: *EAC* Endometrioid Adenocarcinoma, *EBRT* External Beam Pelvic Radiotherapy, *VBT* Vaginal Brachytherapy

As for the 75 NEC patients, there were 7 relapses (9.3%) with DM dominated (*n* = 6, 8.0%). 5 patients only had distant metastasis, 1 patient only had pelvic regional failure, and 1 patient had both vaginal stump failure and distant metastasis.

### Toxicity

Adverse effects (AEs) were recorded on 899 out of 1048 patients who received postoperative RT (Table 3). No radiation-related death occurred. The most common acute and chronic AE was gastrointestinal reactions for patients who had received either VBT alone or EBRT (with or without VBT). In terms of acute reactions, compared with VBT alone, EBRT (with or without VBT) significantly increased the incidence rate of grade 1–2 intestinal, urinary, and hematological AE, and increased the probability of grade 3–4 acute hematological toxicity. As for chronic AE, EBRT significantly increased the rate of grade 1–2 intestinal, urinary, and hematological AEs and edema. There was no significant difference in grade 3–4 late AEs.

**Table 3:** Acute and Choronic Adverse Effects for Patients Treated With VBT alone or EBRT (with or without VBT)

	Patients (*N* = 899)					
	***Early Adverse Effects***			***Late Adverse Effects***		
	VBT alone (***n*** = 499)	EBRT (with or without VBT) (***n*** = 400)	***p***	VBT alone (***n*** = 499)	EBRT (with or without VBT) (***n*** = 400)	***p***
**Adverse Events**	No. (%)	No. (%)		No. (%)		
**GI**			0.000			0.001
0	293 (73.3)	114 (22.8)		358 (89.5)	406 (81.4)	
1–2	107 (26.8)	382 (76.6)		41 (10.3)	92 (18.4)	
3–4	0 (0.0)	3 (0.6)		1 (0.3)	1 (0.2)	
**Urinary Tract**			0.000			0.003
0	376 (94.0)	416 (83.4)		384 (96.0)	453 (90.8)	
1–2	24 (6.0)	82 (16.4)		16 (4.0)	45 (9.0)	
3–4	0 (0.4)	1 (0.6)		0 (0.0)	1 (0.2)	
**Hematological**			0.000			0.005
0	371 (92.8)	271 (54.3)		383 (95.8)	456 (91.4)	
1–2	29 (7.2)	204 (40.9)		15 (3.8)	42 (8.4)	
3–4	0 (0.0)	24 (4.8)		2 (0.5)	1 (0.2)	
**Edema**						0.000
0	/	/		379 (94.8)	428 (85.8)	
1–2	/	/		21 (5.3)	71 (14.2)	
3–4	/	/		0 (0.0)	0 (0.0)	

*Abbreviation*s: *GI* gastrointestinal, *EBRT* External Beam Pelvic Radiotherapy, *VBT* Vaginal Brachytherapy

## Discussion

In this study, we retrospectively analyzed the multi-institutional data of stage I to II EC patients who had received surgery and adjuvant RT from 2003 to 2015 and provided an inclusive overview on survival data, failure pattern, and toxicity of different radiation modalities in different risk groups. This was the first, largest and most detailed introduction to survival efficacy of adjuvant RT for early-stage EC in China thus far.

Along with the results of PORTEC series, GOG (Gynecologic Oncology Group) study [10–15], guidelines were developed [3, 16, 17] that recommended VBT alone for LR, IR and HIR patients as VBT achieved comparable survival outcomes and less toxicity to EBRT, while EBRT (with or without VBT) was recommended for HR stage I and II patients. As for clinical practice, in 2019, the ABS conducted a survey [18] reporting that most (79%) respondents preferred to use adjuvant VBT alone for LR or IR patients and EBRT with (28%) or without VBT (33%) for HR stage I patients. Modh et al. [19] analyzed SEER data and reported that most HR patients like stage II (FIGO 1988) cases were administrated EBRT (60.1%). The results in this study were consistent with the above guidelines and clinical practices elsewhere. However, in Modh’s research, for HR patients like clear-cell/serous histology, most (67.4%) patients received VBT, which was different from our findings.

### Survival

For EAC patients, we compared the efficacy of VBT alone and EBRT (with or without VBT) in different risk groups. As previous studies reported, the locoregional recurrence rate was low for LR, IR and HIR patients. Replacement of VBT with EBRT could not significantly decrease the locoregional failure rate and could not translate into survival benefits [14, 20, 21]. With the upgrade of stage, differentiation and LVSI, the local, regional, and distant recurrence rates increased. As a regional treatment, EBRT could effectively decrease pelvic and vaginal stump relapse rates and indirectly played a role in decreasing distant relapse, ultimately benefiting survival.

When compared to grade 3 EAC, most research demonstrated that NEC had been associated with significantly poorer survival rates [6–8]. For stage I-II disease, 5-OS of uterine papillary serous carcinoma, clear cell carcinoma and grade 3 endometrioid carcinoma was 74, 82, and 86% (*P* < 0.0001) respectively [8]. However, Creasman et al. [22] reported, when diagnosed as stage I, uterine papillary serous carcinoma (5-OS: 72%) and clear cell carcinoma (5-OS: 81%) had no worse survival than G3 endometrioid cancers (5-OS: 76%). With the development of molecular pathology, ESGO / ESTRO / ESP brings molecular characteristics into the prognostic stratification this year. They demonstrated, stage IA p53abn NEC (serial, clear cell, undifferentiated carcinoma, carcinosarcoma, mixed) without MI had good prognosis and was classified into the IR group [4]. In our research, compared with EAC, the prognosis of NEC was slightly worse. Compared with other studies, the survival rate of NEC patients in this study was higher [8, 22, 23]. The reasons were as follows: in this research, mixed cell carcinoma was defined as NEC, while studies had shown that the prognosis of mixed cell carcinoma was better than that of simple NEC [24]. In addition, we did not carry out molecular genomic detection, there might exist p53abn stage IA NEC, which had better prognosis.

### Failure pattern

Chemotherapy could reduce DM rate by eradicating micro-metastasis. For NEC or advanced EAC patients, chemotherapy is necessary. However, for early-stage EAC, the chemotherapy efficacy is controversial. Studies have been conducted to evaluate the efficacy of chemotherapy for HR stage I to II EC patients. Reynaers et al. [25] retrospectively analyzed 116 patients with stage IB-II grade 3 disease and concluded that adjuvant chemotherapy and RT resulted in improved OS compared to RT alone. Smogeli et al. [26] demonstrated that HR EC patients had acceptable vaginal/pelvic control rates after adjuvant chemotherapy alone. However, some studies made opposite conclusions. The PORTECT3 trial [27] showed that chemoradiotherapy did not significantly improve the 5-year failure-free survival compared to RT alone for early-stage HR patients. GOG-0249 [28] also failed to demonstrate the superiority of VBT with chemotherapy versus EBRT among early-stage HR EC patients. Chemotherapy still requires further research before it can be administered to specific populations.

### Toxicity

The VBT dose is confined mostly to the vagina mucosa with less dose to surrounding normal tissues. Hence, the rates of bladder, rectum, intestinal, and bone marrow toxicities are quite low [2, 14]. Studies demonstrated that VBT alone had low rates (range: 0–6.9%) of high-grade complications among which vaginal toxicity was the major one [17]. EBRT not only irradiated the pelvic lymphatic drainage area but also affected the lymphatic drainage of the lower limbs and vulva. In addition, part of the small intestines, rectum and bladder were irradiated. However, the incidence of grade 3–4 acute and chronic AE were low because the doses for organs at risk were still within the tolerance range.

Chemotherapy or EBRT was used mostly for higher risk cases. In this study, the application rate of chemotherapy in EBRT (with or without VBT) group was significantly higher than that in VBT alone group (31.7% vs. 9.0%, *P* = 0.000). As chemotherapy can also cause hematological toxicity, the use of chemotherapy might confound the effect of EBRT on hematological system which weaken the conclusion that EBRT (with or without VBT) increased the probability of acute hematological toxicity.

### Limitations

There were some limitations to this study. As this retrospective study included patients from 2003 and 2015, some of the pathology information was missing (such as amount of LVSI), and some was not included (such as molecular profile). VBT, either as monotherapy or as a boost to EBRT, had a great impact on the vagina due to the high dose per fraction. However, this study did not evaluate vaginal toxicity, which was a limitation. Despite these limitations, this was the first long-term retrospective multicenter study that has investigated the survival outcomes, failure pattern, and toxicity of different postoperative RT modalities in stage I to II EC patients in China.

## Conclusions

EAC accounted for the majority of stage I to II EC patients with different pathological characteristics from NEC. For EAC patients, EBRT (with or without VBT) significantly increased survival compared to that with VBT alone in the HR group. In the LR to HIR groups, VBT alone resulted in comparable survival. Compared with EAC patients, NEC patients had lower OS, DFS, LRFS and DMFS.

## Data Availability

The datasets used and analyzed during the current study are available from the corresponding author upon reasonable request.

## Abbreviations

EAC: Endometrioid adenocarcinoma
NEC: Non-endometrioid carcinoma
EC: Endometrial cancer
LR: Low-risk
IR: Intermediate-risk
HIR: High-intermediate-risk
HR: High-risk
RT: Radiotherapy
EBRT: External beam radiation
VBT: Vaginal brachytherapy
ESMO: European Society for Medical Oncology
ESTRO: European Society for Radiotherapy & Oncology
ESGO: European Society of Gynaecological Oncology
GOG: Gynecologic Oncology Group
WHO: World Health Organization
LVSI: Lymphovascular invasion
FIGO: International Federation of Gynecology and Obstetrics
OS: Overall survival
DFS: Disease free survival
LRFS: Local-regional failure free survival
DMFS: Distant metastasis failure free survival
MI: Myometrial invasion
CTCAE 4.0: Common Terminology Criteria for Adverse Events Version 4.0
SEER: Surveillance, Epidemiology, and End Results Database
AE: Adverse effects
